# A Retrospective Study of Clinical and Radiographic Outcomes and Exploratory Analysis of Implant Failure in Dogs ≤5 kg Treated with Titanium Locking Mini-Plates for Radial and Ulnar Fractures

**DOI:** 10.3390/vetsci13030286

**Published:** 2026-03-18

**Authors:** Alberto Maria Crovace, Marta Guadalupi, Roberta Belvito, Chiara Monopoli, Alejandro Artiles, Eraldo Sanna Passino, Antonio Crovace

**Affiliations:** 1 Department of Veterinary Medicine, University of Sassari, Via Vienna 2, 07100 Sassari, Italy; esp@uniss.it; 2 Department of Precision and Regenerative Medicine and Jonian area (DiMePRe-J), Campus di Medicina Veterinaria, Università Degli Studi di Bari “Aldo Moro”, s.p. per Casamassima km 3, Valenzano, 70010 Bari, Italy; r.belvito@phd.uniba.it (R.B.); cmonopoliroca@phd.uniba.it (C.M.); antonio.crovace@uniba.it (A.C.); 3 Hospital Veterinario Los Tarahales, Ctra. de los Tarahales, 15, 35013 Las Palmas, Spain; a.artiles@hvtarahales.es

**Keywords:** toy-breed dogs, fracture stabilization, locking plate fixation, distal fragment length, implant failure

## Abstract

Radial and ulnar fractures in toy-breed dogs are associated with a high risk of complications due to limited bone stock and soft tissue coverage. The aim of this retrospective study was to evaluate the postoperative clinical and radiographic outcomes in dogs undergoing radial and ulnar fracture stabilization with a titanium locking mini-plate system. Medical records and radiographs of toy-breed dogs weighing ≤5 kg treated between 2020 and 2025 were reviewed. Twenty-six dogs met the inclusion criteria. Radiographic union after primary fixation was observed in 92.9% of fractures. Implant failure occurred in 7.1% of cases and was successfully addressed with revision surgery, after which fracture healing was achieved. At final follow-up, limb function was graded as excellent in all dogs, including those requiring revision surgery. A shorter distal fragment length was significantly associated with implant failure, whereas implant length and proximal fragment length showed no significant association. These findings indicate that titanium locking mini-plates were associated with favorable clinical and radiographic outcomes. Distal fragment dimensions may influence the risk of implant failure and should be considered during surgical planning. However, the small number of implant failure events limits the robustness of statistical inferences, and the absence of a control group prevents direct comparison with alternative fixation techniques.

## Simple Summary

Radial and ulnar fractures are common in small dogs and can be difficult to treat because their bones are thin and fragile. Surgical stabilization using small bone plates is frequently required, but complications such as implant failure or delayed healing may occur. This study reviewed the medical records of dogs weighing 5 kg or less that were treated for these fractures using a titanium locking mini-plate system. In most cases, fractures healed successfully after surgery, and at the final follow-up, all dogs regained normal limb function. These findings suggest that the titanium locking plates used in this study were associated with stable fracture fixation in toy-breed dogs.

## 1. Introduction

Radial and ulnar fractures are among the most common long-bone injuries in dogs, accounting for approximately 18% of all fractures in this species [1,2,3,4]. Toy-breed dogs are disproportionately affected, largely due to specific biomechanical characteristics. Although the cortical bone thickness is proportionally greater in small dogs, the overall cross-sectional geometry results in a reduced moment of inertia. Consequently, when subjected to similar mechanical loads, the distal radius in toy breeds experiences higher stress concentrations, predisposing it to fracture [5].

Fracture management in dogs weighing ≤5 kg remains clinically challenging. Bone plate fixation, while widely used, has historically been associated with relatively high complication rates in this population, including delayed union, nonunion, implant failure, and malalignment [2,6]. However, limited distal fragment size, reduced bone stock, and compromised local vascularity are considered important contributing factors that may adversely affect fixation stability and fracture healing [7].

Various surgical options have been described for radial and ulnar fractures in small dogs, including open or minimally invasive plate fixation [8,9], circular or linear external fixation [10,11], intramedullary techniques, and regenerative approaches, including bone grafting procedures, scaffold-based biomaterials, and osteoinductive growth factors [12,13,14,15,16,17].

More recently, locking plate systems have been introduced to improve construct stability, particularly in cases with limited bone stock. Locking plate constructs act as fixed-angle internal fixators: stability is generated primarily by the locked screw–plate interface rather than by frictional compression between the plate and bone. As a result, construct behavior can be tuned by screw number and distribution, plate working length, and bridge span, while limiting the need for plate–bone compression, an approach that may help preserve periosteal perfusion and supports biologic fracture healing [18,19].

Swepson et al. [20] reported favorable clinical and radiographic outcomes using 1.5 mm locking plates for radial and ulnar fractures in dogs, confirming the biomechanical reliability of locking plate constructs in small patients. However, fixation-related complications remain clinically relevant, especially in fractures characterized by short distal fragments.

Despite advances in implant technology, limited information is available regarding the potential influence of fracture morphology and implant-related variables on fixation outcome in dogs weighing ≤5 kg. In particular, the role of fragment length and implant length in determining system stability has not been fully clarified.

The primary objective of this retrospective study was to comprehensively evaluate radiographic morphometric variables, implant characteristics, and postoperative radiographic and clinical outcomes in toy-breed dogs weighing ≤5 kg undergoing radial and ulnar fracture stabilization with a titanium locking mini-plate system.

Furthermore, given the high rate of fixation-related complications reported in toy-breed dogs with radial and ulnar fractures, the present study aimed to investigate whether morphometric and implant-related variables may be related to the occurrence of implant failure.

## 2. Materials and Methods

### 2.1. Patients

This retrospective study involved a review of medical records from the Veterinary Surgical Unit of DiMePre-J, University of Bari “Aldo Moro,” identifying all dogs that underwent surgical repair of radial and ulnar fractures between 2020 and 2025. For each case, data collected from the medical records included: breed, sex, age (expressed in months), body weight (expressed in kg), preoperative radiographic evaluation, implant type, postoperative fracture alignment, postoperative complications, and follow-up radiographic evaluation.

Written informed consent was obtained from all dog owners at the time of hospital admission. The consent form provided detailed information regarding the planned surgical procedure and included authorization for the use of anonymized clinical and imaging data for research purposes.

### 2.2. Inclusion and Exclusion Criteria

Cases were considered eligible for inclusion in this retrospective study if they involved dogs weighing 5 kg or less that underwent surgical repair of a complete, recent, and closed radial and ulnar fracture using the titanium locking mini-plate system, with fracture stabilization performed by open reduction and internal fixation and the plate applied to the cranial surface of the radius. Dogs re-presenting with a contralateral antebrachial fracture, as well as cases undergoing surgical revision, were also included. Moreover, only cases with complete and retrievable medical records were included in the analysis. Cases were excluded if medical records were incomplete or if pre- or post-operative radiographs or one or more required follow-up radiographic examinations were missing.

### 2.3. Implant Types

Osteosynthesis was performed using a commercially pure (CP) grade 4 titanium locking mini-plate system intended for small animal fracture stabilization (Biosurgex S.L., Las Palmas, Spain). The system includes anatomically adaptable plates available in two thicknesses (1.5 mm and 2.0 mm). In the present study, 2.0 mm plates were selected for all fractures. According to fracture configuration and fragment size, straight plates (including cuttable plates) or T-shaped plates were selected. Locking screws of 1.5 mm or 2.0 mm diameter were applied depending on bone size and fragment dimensions.

In all cases, plate selection was guided by the principle of achieving adequate fixation of both fragments while respecting fragment length and bone geometry. Particular attention was paid to ensuring a minimum of two locking screws per fragment whenever feasible and to avoiding excessive plate length relative to total radial length. The final implant configuration was determined intraoperatively based on fragment stability after reduction and the ability to obtain secure screw purchase within the available bone stock.

### 2.4. Stabilization Method

**Figure 1:**
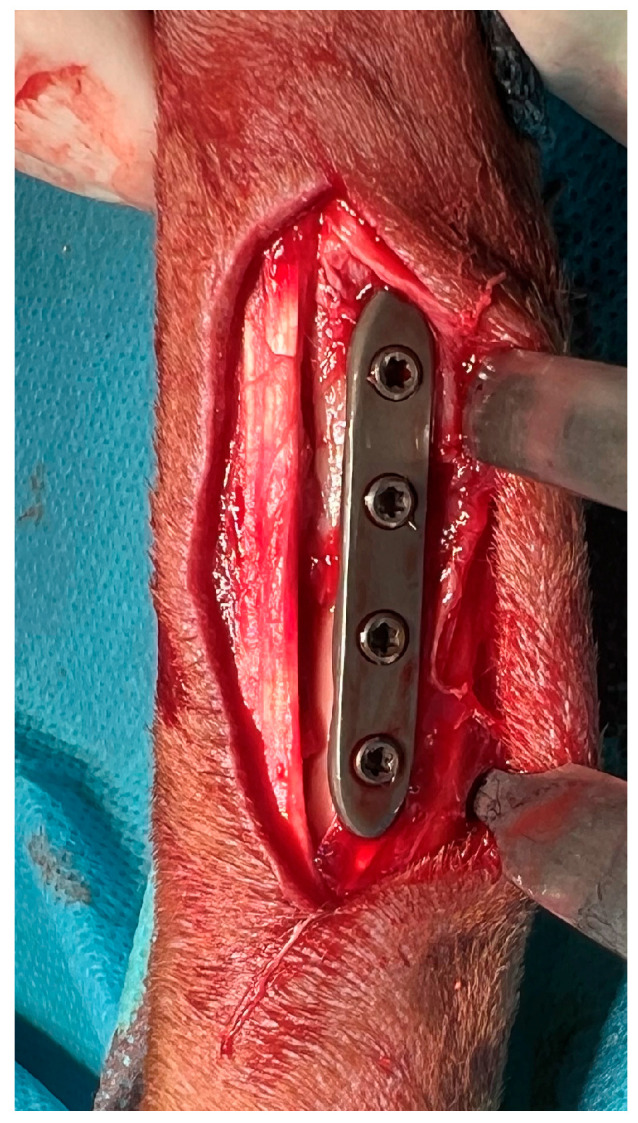
Intraoperative view of plate placement during radial fracture stabilization, showing the titanium locking plate positioned on the cranial surface of the radius during open reduction and internal fixation of a radial and ulnar fracture.

All procedures were performed by two experienced surgeons via an open surgical approach, with consensus reached in cases of discrepancy. After exposure of the fracture site, meticulous soft tissue handling was maintained to preserve the periosteal blood supply. Fracture reduction was achieved using manual traction or reduction forceps. The plate was positioned on the cranial surface of the radius in all cases, and contouring was performed as needed to match the natural curvature of the bone (Figure 1). Plate configuration was selected based on fracture location, fragment size, and the ability to achieve adequate screw distribution. Straight plates were used for simple mid-diaphyseal fractures in which at least two screws per fragment could be securely positioned along the longitudinal axis of the radius. T-shaped plates were selected for distal epiphyseal or metaphyseal fractures requiring a broader distal screw spread to enhance angular stability. Cuttable plates were chosen when intraoperative customization of plate length or screw spacing was necessary, particularly in cases with unusual fracture configurations or atypical bone geometry. When cuttable plates were shortened intraoperatively, no further modification of the plate edges was necessary, as the system is designed to permit intraoperative cutting without generating sharp or irregular margins requiring additional contouring.

After implant placement, the surgical site was irrigated with sterile saline to remove debris and blood clots. Closure was performed in multiple anatomical layers: the periosteal and muscular layers were closed using absorbable monofilament sutures in a simple continuous pattern, followed by subcutaneous closure to reduce dead space. The skin was closed with either absorbable interrupted sutures or staples, according to surgeon preference.

### 2.5. Radiographic Evaluations

Standard orthogonal radiographic projections, mediolateral and craniocaudal views, were obtained for each dog using a digital radiographic generator (ARIA450 Digital, Foschi, Italy). Radiographic measurements and image assessments were performed using Horos, an open-source DICOM viewer based on OsiriX 5.8 (Horos™, Horos Project, Geneva, Switzerland), to ensure consistent and accurate evaluation across all cases. All radiographic evaluations were independently performed by two experienced surgeons and one PhD student, with consensus reached in cases of discrepancy.

#### 2.5.1. Preoperative Radiographic Assessment

**Figure 2:**
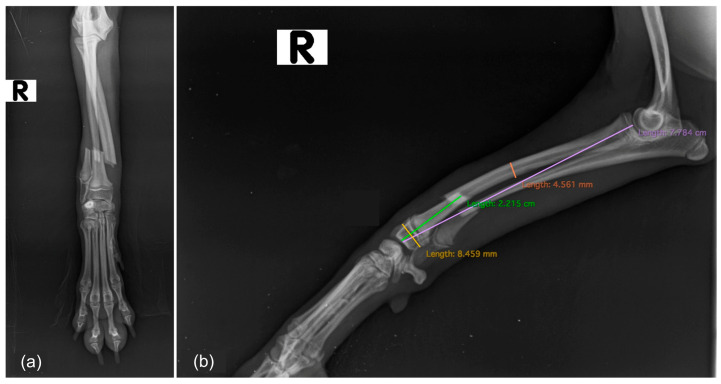
Preoperative radiographic assessment included standard orthogonal projections: (**a**) craniocaudal view, and (**b**) mediolateral view showing the measurements of the morphometric parameters evaluated in the study.

Preoperative radiographs were reviewed to characterize the fracture morphology and collect all relevant morphometric parameters (Figure 2). In particular, the affected limb, fracture location (proximal, mid or distal diaphyseal third), and fracture configuration (transverse, short or long oblique, spiral, or comminuted) were recorded. In skeletally immature dogs, the status of the distal radial physis was also assessed. Quantitative measurements of morphometric parameters included proximal fragment length, distal fragment length, total radius length, width of both the mid-diaphysis and the distal epiphysis, all expressed in mm, and the distal fragment–to–total radius length ratio. All morphometric parameters were measured on the mediolateral radiographic projection.

#### 2.5.2. Postoperative Radiographic Assessment

Immediate postoperative radiographs were reviewed to evaluate fracture reduction and implant positioning. Translational malalignment was quantified as the ratio between maximal displacement and bone width at the fracture site. Postoperative alignment was then categorized into established percentage displacement groups: <1% (anatomical reduction), <5% (near-anatomical reduction), <10%, <25%, and >25% [21]. Additional implant-related parameters were recorded, including plate type, plate length (mm), and the number of locking screws distributes within the proximal and the distal fragments.

#### 2.5.3. Follow-Up Radiographic Assessment

Radiographic assessments performed at 30, 60, and 90 days after surgery were retrospectively evaluated in order to evaluate the fracture healing and the union progression, using the standardized radiographic scoring system developed by the International Society of Limb Salvage (ISOLS) [21,22] that defines “union” as the fusion of the fracture line, the presence of bridging callus, or the disappearance of the fracture line observed on at least one orthogonal radiographic view. Based on ISOLS scoring system, healing was graded as follows: 1 = “poor union”, defined as less than 25% healing with no evidence of callus formation, 2 = “fair union”, corresponded to 25–50% healing, 3 = “good union”, represented more than 50% but up to 75% radiographic healing, and 4 = “excellent union”, assigned when more than 75% of the fracture showed radiographic evidence of healing.

### 2.6. Postoperative Complications and Clinical Outcome

Postoperative complications were recorded and classified as minor or major according to the criteria described by Cook et al. [23]. Minor complications were managed conservatively, whereas major complications required surgical intervention or adversely affected the expected clinical outcome.

Overall limb function was assessed at follow-up through clinical examination performed by the attending surgeon and based on visual gait assessment at walk and trot. Limb function was categorized as “excellent” (return to normal function without observable lameness), “good” (mild intermittent lameness without functional impairment), “fair” (frequent mild to moderate lameness), or “poor” (persistent moderate to severe lameness affecting daily activity) [21].

### 2.7. Statistical Evaluation

Statistical analysis was performed with a free statistical software Jamovi (version 2.7.12; The Jamovi project, Sydney, Australia, https://www.jamovi.org). Data distribution was assessed using the Shapiro–Wilk test. Continuous variables were summarized as mean ± standard deviation (SD). Categorical variables were expressed as frequencies and percentages.

Descriptive statistical analysis was conducted for demographic variables (breed, sex, age, and body weight), for fracture morphology, for morphometric parameters, and for all radiographic measurements, including proximal fragment length, distal fragment length, total radius length, distal fragment–to–total radius length ratio, distal epiphysis width, mid-diaphysis width, implant length, and number of screws. Moreover, the immediate postoperative alignment categories, the radiographic healing, the postoperative complications, and the overall limb function were analyzed descriptively.

Welch’s one-way ANOVA analysis was performed to explore the association between implant failure and three variables: implant length, proximal fragment length, and distal fragment length. Effect sizes (η^2^) were calculated to quantify the magnitude of group differences.

A binary logistic regression analysis was performed to evaluate the association between total radius length (mm) and the occurrence of implant failure. Implant failure was treated as a dichotomous dependent variable (yes/no), while total radius length was included as a continuous independent predictor. The model was fitted using maximum likelihood estimation. Model adequacy was assessed using the likelihood ratio test, and goodness-of-fit was quantified using the pseudo–R^2^ statistic (McFadden). Regression coefficients (β), standard errors, Wald statistics, odds ratios (OR), and corresponding 95% confidence intervals were calculated.

A *p* < 0.05 was considered statistically significant for all analyses.

## 3. Results

### 3.1. Patients

A total of 26 dogs met the inclusion criteria and were enrolled in the study. The causes of fractures reported in medical records were a jump, a fall, or a drop from a height. The study population consisted of Toy Poodles (n = 8), Pinschers (n = 6), mixed-breed dogs (n = 5), Pomeranians (n = 3), Poodles (n = 2), Yorkshire Terriers (n = 1), and Spitz (n = 1). Of the enrolled dogs, 15 were females and 11 were males. The mean ± SD age was 18.21 ± 15.75 months (range: 4–72 months), whereas the mean body weight ± SD was 3.11 ± 0.93 kg (range: 1.20–4.50 kg).

### 3.2. Fractures Description and Preoperative Radiographic Assessment

A total of 28 fractures were treated: 11 involved the right thoracic limb and 17 the left thoracic limb. Notably, two fractures occurred in the same dog, which sustained separate injuries affecting each forelimb at different times. Regarding anatomical location, 21 fractures affected the distal third of the radial diaphysis, 19 of which were transverse, whereas 2 were short oblique. The remaining 7 fractures involved the mid-diaphyseal third of the radius. No proximal diaphyseal fractures were observed in this cohort.

Moreover, in 6 dogs, the distal radial physis was still open; however, the implants did not bridge the physis in any case.

Descriptive statistics for all radiographic morphometric variables are reported in Table 1. The mean ± SD proximal fragment length was 57.071 ± 16.824 mm, the mean ± SD distal fragment length measured 24.091 ± 10.777 mm, the total radius length had a mean ± SD value of 81.162 ± 17.480 mm, the distal fragment–to–total radius length ratio showed a mean ± SD of 0.300 ± 0.120, the mean ± SD width of the distal epiphysis was 6.625 ± 2.261 mm, while the mean ± SD mid diaphyseal width was 4.309 ± 1.494 mm.

Normality of these variables was assessed using the Shapiro–Wilk test. Proximal fragment length and the distal fragment–to–total radius length ratio did not significantly deviate from a normal distribution (*p* = 0.151 and *p* = 0.090, respectively). In contrast, distal fragment length, total radius length, distal epiphyseal width, and mid-diaphyseal width showed statistically significant deviations from normality (all *p* < 0.05).

### 3.3. Implant Types

Fracture stabilization was achieved using a titanium locking mini-plate system. A total of 11 straight locking plates (including 6 cuttable versions) and 13 T-shaped locking plates (including 7 cuttable configurations) were used. All fractures were repaired using 2 mm plates. The mean ± SD plate length was 38.18 ± 13.01 mm (range: 20–70 mm), whereas the mean ± SD number of screws per plate was 4.50 ± 0.75 (range: 4–6 screws). Screw diameter selection varied according to fragment size: 1.5 mm locking screws were used in 12 cases, whereas 2.0 mm locking screws were employed in 19 cases. Moreover, at least two locking screws were inserted into the distal fragment in all cases.

### 3.4. Postoperative and Follow-Up Radiographic Assessment

Immediate postoperative radiographs demonstrated that fracture reduction and implant positioning were satisfactory in all 28 cases. Based on established displacement categories, all fractures achieved either anatomical reduction (<1%) or near-anatomical reduction (<5%), with no cases falling into the <10%, <25%, or >25% malalignment groups.

**Figure 3:**
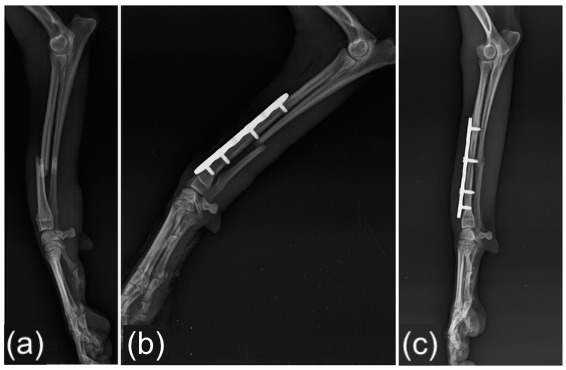
Radiographic images of a radio-ulnar fracture treated with a 2.0 mm straight locking plate with six holes and four screws. (**a**) Preoperative mediolateral radiograph showing the fracture configuration. (**b**) Immediate postoperative radiographs demonstrating satisfactory fracture reduction and implant positioning. (**c**) Radiographic follow-up at two months showing fracture healing consistent with excellent union according to the ISOLS scoring system.

**Figure 4:**
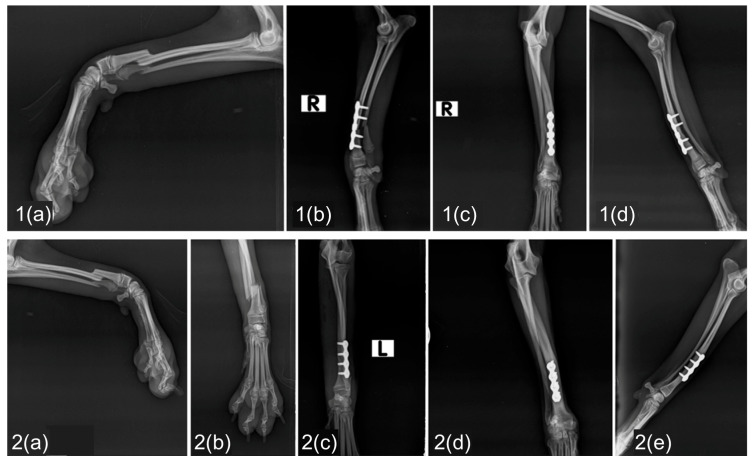
Bilateral distal radial and ulnar fractures treated with cuttable locking plates. Radiographic sequence of bilateral distal-third radial and ulnar fractures stabilized using 2.0 mm cuttable locking plates. (**1.a**) Preoperative radiographs of the first fracture. (**1.b**) Immediate postoperative radiographs demonstrating satisfactory fracture reduction and (**1.c**,**d**) follow-up at two months showing satisfactory bone healing consistent with ISOLS excellent union of the first limb. (**2.a**,**b**) Preoperative radiographs of the contralateral fracture. (**2.c**) Immediate postoperative radiographs demonstrate satisfactory fracture reduction. (**2.d**,**e**) Radiographic follow-up at two months demonstrating satisfactory bone healing consistent with ISOLS excellent union.

At the 60-day follow-up after definitive stabilization (primary fixation in 26 fractures and revision surgery in 2 fractures), radiographic signs of union (fusion of the fracture line, bridging callus formation, or fracture line resolution) were observed in all 28 cases. Among these, 21 fractures achieved an ISOLS score of 4 (excellent union), while 7 fractures received a score of 3 (good union) (Figure 3 and Figure 4).

### 3.5. Postoperative Complications and Clinical Outcome

**Figure 5:**
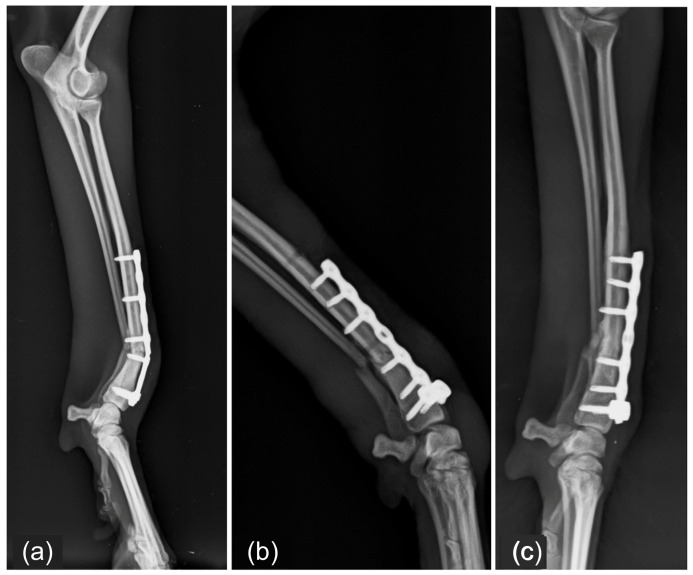
Major implant-related complications and revision surgery. (**a**) Postoperative mechanical failure of a cuttable T-shaped locking plate with eight holes and six screws, classified as implant failure. (**b**) Revision surgery with replacement of the failed implant using a cuttable T-shaped locking plate with eight holes and seven 2.0 mm locking screws. (**c**) Radiographic follow-up three months after plate replacement, showing satisfactory implant positioning and progression of fracture healing.

**Figure 6:**
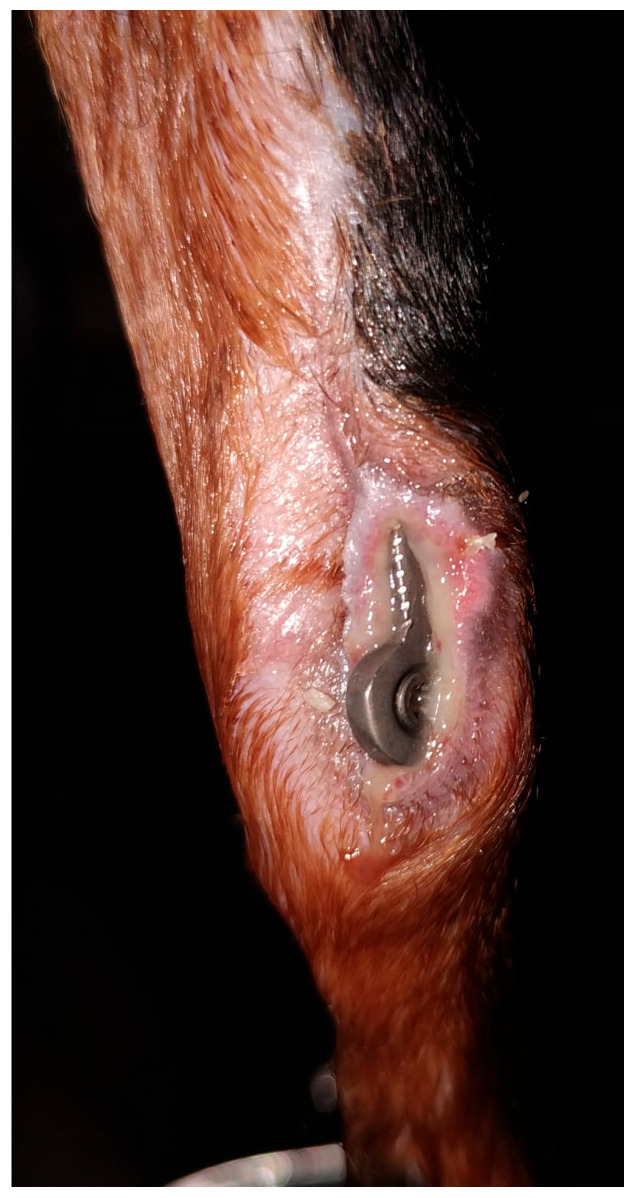
Major non–mechanical implant-related complication at postoperative follow-up. Postoperative exposure of a straight T-shaped locking plate applied to a distal radial fracture due to soft-tissue dehiscence, observed during the postoperative follow-up period.

No minor complications were observed in any of the treated cases during the postoperative period. However, major complications occurred in 3 cases (10.7%). Implant failure was recorded in 2 cases (7.1%), both involving postoperative mechanical breakage of a T-shaped plate (Figure 5); the implant was subsequently replaced with a cuttable T plate featuring eight holes and seven locking screws. In 1 case (3.6%), a T-shaped plate applied to a distal radial fracture became exposed due to soft-tissue dehiscence (Figure 6); this case was managed by surgical removal of the implant.

Importantly, overall limb function was graded as excellent in 28 cases, including those that underwent revision, starting from the 30-day postoperative follow-up and maintained throughout subsequent evaluations.

### 3.6. Association of Morphometric and Implant Length Variables on Implant Failure

Implant failure occurred in 2 fractures (7.1%). The remaining 26 fractures (92.9%) healed without implant failure.

Welch’s one-way ANOVA was performed to assess whether morphometric and implant-related variables differed between cases with (n = 2) and without (n = 26) implant failure. Each morphometric and implant-related variable was independently compared between the failure and non-failure groups.

Proximal fragment length did not differ significantly between the two conditions (F = 0.739, df = 1, 1.11, *p* = 0.536). Implant length also showed no statistically significant association with implant failure (F = 5.086, df = 1, 1.47, *p* = 0.197). In contrast, distal fragment length differed significantly between dogs with implant failure and those with successful fixation (F = 6.292, df = 1, 10.76, *p* = 0.029). Effect sizes (η^2^) were calculated to quantify the magnitude of the association between morphometric and implant length variables and implant failure. Proximal fragment length showed a moderate effect (η^2^ = 0.40), whereas distal fragment length also demonstrated a moderate effect (η^2^ = 0.37), consistent with its statistically significant association with implant failure. Implant length yielded a large η^2^ estimate (η^2^ = 0.78). However, given the limited number of implant failure events, these effect size estimates, particularly for implant length, should be interpreted cautiously, as effect size measures may be unstable in small samples.

These results were summarized in Table 2.

**Figure 7:**
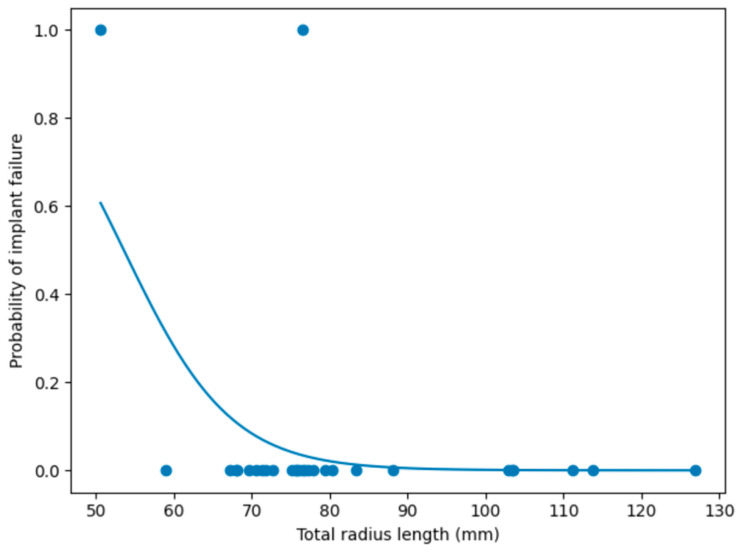
Logistic regression analysis of implant failure in relation to total radius length. Scatter plot showing individual observations (implant failure: yes/no) and the fitted logistic regression curve illustrating the relationship between total radius length (mm) and the estimated probability of implant failure. Shorter total radius length was associated with a higher probability of implant failure, although the association did not reach statistical significance.

Binary logistic regression analysis was performed to investigate the association between total radius length and the occurrence of implant failure. The overall model was statistically significant (likelihood ratio test, *p* = 0.049) and accounted for approximately 26.6% of the variance in implant failure (McFadden pseudo–R^2^ = 0.266). Total radius length showed a negative association with implant failure (β = −0.146), indicating that shorter radii were associated with an increased likelihood of implant failure. The corresponding odds ratio was 0.86, suggesting that for each 1 mm increase in total radius length, the odds of implant failure decreased by approximately 14%. However, this association did not reach statistical significance (*p* = 0.100). The relationship between total radius length and implant failure was illustrated in Figure 7, which showed a decreasing probability of implant failure with increasing radius length.

## 4. Discussion

The aim of this retrospective study was to evaluate clinical and radiographic outcomes in dogs weighing ≤5 kg undergoing radial and ulnar fracture stabilization with a titanium locking mini-plate system, and to explore whether morphometric and implant-related variables were associated with implant failure. The findings of this study provide data addressing these objectives and provide preliminary evidence regarding the relationship between morphometric variables and implant failure.

The locking mini-plate system allowed stable fracture fixation and consistent fracture healing following primary surgery in 92.9% of cases, with a high proportion of excellent functional outcomes at final follow-up. These findings align with previously reported outcomes for internal fixation of radial fractures in small-breed dogs, where union rates ranging from 70% to 95% [24,25,26] and complication rates up to 68% [27] have been described. Although in our cohort implant failure occurred in two cases (7.1%), both were successfully managed with revision surgery, resulting in eventual fracture union.

The implant system employed in this study includes plates available in different configurations and thicknesses compatible with small-diameter locking screws, allowing adaptation to the limited bone stock that characterizes the distal radius in dogs ≤ 5 kg. The availability of cuttable plates represents a further advantage, as it enables intraoperative customization of plate length and screw distribution according to fracture configuration and fragment size, which is particularly relevant in distal diaphyseal fractures. Such design characteristics may facilitate screw placement and construct stability in fractures with short distal fragments, a recognized challenge in this population [20].

The locking mechanism of the system provides angular stability between the screw and the plate, reducing reliance on plate–bone friction, potentially preserving periosteal blood supply, a factor considered important for fracture healing, as previously described [18,19]. This feature is especially advantageous in small-breed dogs, where excessive compression at the plate–bone interface may compromise vascularization and delay healing. The high rate of anatomical or near-anatomical reduction observed in the present study, together with the favorable radiographic healing outcomes, suggests that stable fixation can be achieved with this implant system while potentially minimizing disruption of the local biological environment.

An additional important consideration is the implant material itself. The plates are manufactured from commercially pure (CP) grade 4 titanium, a material widely employed in orthopedic applications due to its several advantages. CP grade 4 titanium exhibits a lower elastic modulus that more closely approximates that of cortical bone, potentially reducing stress shielding and promoting more physiological load transfer during fracture healing [28,29]. Moreover, titanium’s excellent corrosion resistance and biocompatibility, resulting from the formation of a stable titanium oxide layer, are associated with reduced inflammatory response and favorable tissue integration [30,31]. These material properties may be particularly advantageous in small-breed dogs, where limited soft-tissue coverage and compromised vascularity increase the risk of implant-related complications.

A key finding of the present study was the statistically significant association between distal fragment length and implant failure, as demonstrated by Welch’s one-way ANOVA. Dogs that experienced implant failure had shorter distal fragments compared with cases with successful fixation. This finding suggests that reduced distal bone stock may be mechanically relevant, as limited screw distribution in short distal fragments could contribute to micromovements or increased stress concentration [7,20]. From a biomechanical standpoint, a shorter distal fragment may compromise fixation stability through several mechanisms. Reduced fragment length restricts distal screw placement and decreases the available bone stock for adequate screw purchase [21]. In toy-breed dogs, where bone diameter and cortical area are already limited, diminished screw engagement may reduce resistance to pull-out forces and torsional loads. Furthermore, a shorter distal segment increases the effective lever arm acting on the plate–screw construct during weight bearing, potentially amplifying bending moments at the fracture site [3]. Even in locking constructs, where angular stability reduces reliance on plate-to-bone compression, adequate distal screw distribution remains critical to withstand cyclic loading and torque. Therefore, limited distal fragment length may biomechanically predispose to construct instability, particularly in small-breed dogs characterized by reduced moment of inertia and higher stress concentration [20].

Conversely, neither proximal fragment length nor implant length showed a statistically significant association with implant failure, although implant length demonstrated a large effect size, likely influenced by the small number of failure cases. These findings indicate that fixation stability in distal radial fractures is more strongly influenced by fragment geometry and screw purchase than by overall plate length [18,32].

The logistic regression analysis further supported this interpretation, revealing a negative association between total radius length and implant failure, suggesting that shorter radii may be inherently more susceptible to mechanical complications. Although this association did not reach statistical significance, the overall model explained a meaningful proportion of variance in implant failure, highlighting the potential clinical relevance of morphometric parameters in preoperative planning.

The present study has several limitations that should be acknowledged when interpreting the results. First, the sample size was relatively small, and only a limited number of implant failures were observed. This constraint reduces statistical power, particularly for multivariable analyses, and may explain why some associations, such as those involving implant length and total radius length, did not reach statistical significance despite showing moderate to large effect sizes. Moreover, the inferential statistical analyses performed should be considered exploratory in nature. The relatively small sample size and the low number of implant failure events increase the potential instability of effect size calculations. Further prospective studies, including a larger number of implant failure cases, are required to validate these preliminary observations and to more robustly assess the potential role of distal fragment length as a determinant of fixation stability. Second, radiographic assessments, including fracture healing and alignment scoring, were based on retrospective image review, which may be subject to observer-related variability despite being performed by trained evaluators. Third, the retrospective nature of the study limited the ability to standardize surgical decision-making, including implant selection, plate configuration, and screw number and distribution, which were determined on a case-by-case basis according to surgeon preference and fracture characteristics. Additionally, the heterogeneity of the study population in terms of age and breed may have introduced biological variability that could not be fully accounted for. Furthermore, postoperative limb function assessment was based on clinical and subjective evaluation, without the use of objective kinetic or kinematic gait analysis. The lack of standardized objective functional outcome measures should therefore be considered when interpreting the reported functional results.

Finally, the absence of a control group or direct statistical comparison with alternative fixation techniques precludes definitive conclusions regarding the relative advantage of the implant system evaluated in this study. Prospective, controlled studies with larger sample sizes are therefore warranted to validate these findings and further clarify the biomechanical and clinical factors influencing fixation success in toy-breed dogs.

Overall, the combination of angularly stable fixation, modular plate design, and favorable material properties likely contributed to the generally positive clinical and radiographic outcomes observed in this cohort. Although implant-related complications did occur in a small number of cases, these were limited and manageable, and all dogs ultimately achieved excellent limb function. Taken together, these findings suggest that titanium locking mini-plate systems such as the Biosurgex implants may represent a viable fixation option for radial and ulnar fractures in toy-breed dogs. Moreover, due to the retrospective design and the absence of a control group, direct comparisons with alternative fixation techniques cannot be established based on the present study.

However, surgeons should carefully evaluate distal fragment dimensions during preoperative planning, as extremely short distal fragments often represent the critical point of fixation and may predispose to mechanical failure, even when modern locking implants are used.

## 5. Conclusions

This retrospective study evaluated clinical and radiographic outcomes in dogs weighing ≤5 kg undergoing radial and ulnar fracture stabilization with a titanium locking mini-plate system. Primary fracture union was achieved in most cases, and implant failure was infrequent and successfully managed. Distal fragment length emerged as a potentially relevant mechanical factor associated with implant failure. These findings provide additional clinical insight into fracture stabilization in small-breed dogs; however, further controlled studies are warranted to confirm these observations.

## Figures and Tables

**Table 1:** Descriptive statistics and Shapiro–Wilk normality test for radiographic morphometric variables. Reported values include mean, median, standard deviation (SD), interquartile range (IQR), minimum, maximum, and Shapiro–Wilk test statistic (W) with corresponding *p*-value. Proximal fragment length and the distal fragment–to–total radius length ratio did not significantly deviate from normality (*p* = 0.151 and *p* = 0.090, respectively), whereas distal fragment length, total radius length, distal epiphyseal width, and mid-diaphyseal width showed significant deviations from normality (all *p* < 0.05).

							Shapiro–Wilk	
	Mean	Median	SD	Interquartile Range (IQR)	Minimum	Maximum	W	*p*
Proximal fragment length (mm)	57.071	54.515	16.824	12.870	27.320	101.600	0.945	0.151
Distal fragment length (mm)	24.091	22.165	10.777	8.900	8.700	49.720	0.873	0.003
Total radius length (mm)	81.162	76.295	17.480	13.525	50.610	126.900	0.885	0.005
Distal fragment to total radius length ratio	0.300	0.286	0.120	0.114	0.107	0.641	0.937	0.090
Distal epiphysis width (mm)	6.625	6.840	2.261	1.900	1.010	9.980	0.856	0.001
Mid diaphysis width (mm)	4.309	4.000	1.494	1.625	2.530	7.750	0.886	0.005

**Table 2:** Summary of the Welch’s ANOVA comparing implant length, proximal fragment length, and distal fragment length between cases that experienced implant failure (n = 2) and those with successful fixation (n = 26). The table reports F-values, degrees of freedom (df_1_ and Welch-corrected df_2_), *p*-values, and effect sizes (η^2^). Distal fragment length was the only variable showing a statistically significant difference (*p* < 0.05), with a moderate effect size.

Variable	F-Values	df_1_	df_2_	*p*-Values	η^2^
Proximal fragment length (mm)	0.739	1	1.11	0.536	0.4
Distal fragment length (mm)	6.292	1	10.76	0.029	0.37
Implant length (mm)	5.086	1	1.47	0.197	0.78

## Data Availability

The original contributions presented in this study are included in the article. Further inquiries can be directed to the corresponding author.
